# Genomic Islands in the Full-Genome Sequence of an NAD-Hemin-Independent Avibacterium paragallinarum Strain Isolated from Peru

**DOI:** 10.1128/MRA.00118-19

**Published:** 2019-04-18

**Authors:** Luis Tataje-Lavanda, Ángela Montalván, Ricardo Montesinos, Vladimir Morales-Erasto, Mirko Zimic-Peralta, Manolo Fernández-Sánchez, Manolo Fernández-Díaz

**Affiliations:** a Laboratorios de Investigación y Desarrollo, FARVET, Chincha Alta, Ica, Peru; b Centro de Investigación y Estudios Avanzados en Salud Animal, Facultad de Medicina Veterinaria y Zootecnia, Universidad Autónoma del Estado de México, Toluca, Mexico; c Laboratorio de Bioinformática y Biología Molecular, Facultad de Ciencias, Universidad Peruana Cayetano Heredia, Lima, Peru; University of California, Riverside

## Abstract

Here, we report the full-genome sequence of an NAD-hemin-independent Avibacterium paragallinarum serovar C-2 strain, FARPER-174, isolated from layer hens in Peru. This genome contained 12 potential genomic islands that include ribosomal protein-coding genes, a *nadR* gene, hemocin-coding genes, sequences of fagos, an *rtx* operon, and drug resistance genes.

## ANNOUNCEMENT

Avibacterium paragallinarum is the etiologic agent of infectious coryza, an acute respiratory disease of chickens, which is globally distributed and causes serious economic losses in the poultry production industry. It is a Gram-negative, nonmotile, capsulated, facultative anaerobe belonging to the family *Pasteurellaceae* and is classified in 9 serovars distributed in 3 serogroups (A, B, and C) (1, 2). The study of its genome and virulence factors (hemagglutinin antigen, capsule, lipopolysaccharide, and RTX toxin) is important to better understand the pathogenesis of infectious coryza (3–6). Usually, virulence factor-coding genes are located in genomic islands (GIs) comprising clusters of genes suspected to have a horizontal origin (integrons, transposons, integrative and conjugative elements, and prophages) (7–9).

The strain FARPER-174 was isolated from nasal secretions of the paranasal sinuses of layer hens from an infectious coryza outbreak from the central rainforest region of Peru during 2015 and was grown in blood culture medium with X-(hemin) and V-(NAD) factors (10). FARPER-174 was subsequently cultured in brain heart infusion (BHI) agar without any factors at 37°C in a candle jar for 24 hours. The strain was identified as *A. paragallinarum* serovar C-2 using the morphology of the colony, biochemical tests (catalase test, oxidase test, urease test, peptone test, and carbohydrate fermentation, such as that of maltose, mannitol, lactose, and sorbitol) (11), specific PCR (12), and hemagglutination inhibition (HI) (13). The disk diffusion test (14) revealed resistance to colistin, ampicillin, sulfamethoxazole-trimethoprim, penicillin, neomycin, lincomycin, oxacillin, enrofloxacin, and gentamicin. The strain was susceptible to amoxicillin-clavulanic acid, doxycycline, streptomycin, florfenicol, tiamulin, levofloxacin, and gatifloxacin.

DNA was extracted from the fresh bacterial culture (500 ml of BHI without factors at 37°C with agitation at 180 rpm for 24 hours) centrifuged at 5,000 *× g* for 15 min. The pellet was resuspended in phosphate-buffered saline (PBS) for DNA isolation using the phenol-chloroform protocol (15). The genome was sequenced on the PacBio RS II platform (Pacific Biosciences, CA) using P6-C4 chemistry and assembled with Hierarchical Genome Assembly Process (HGAP) version 3.0 (16) with default parameters by Macrogen, Inc. (South Korea). A total of 92,198 reads (average length, 8,615 bp; *N*_50_, 12,231 bp) generated a closed circular genome of 2,425,949 bp, with a G+C content of 40.87% and 147× average depth of coverage, and no plasmids.

The FARPER-174 strain genome was annotated with the NCBI Prokaryotic Genome Automatic Annotation Pipeline (PGAAP v4.7) (17). Some hypothetical proteins were similar to coding DNA sequences (CDSs) of *A. paragallinarum* previously published in GenBank; the annotations were, therefore, manually improved in this version.

**FIG 1:**
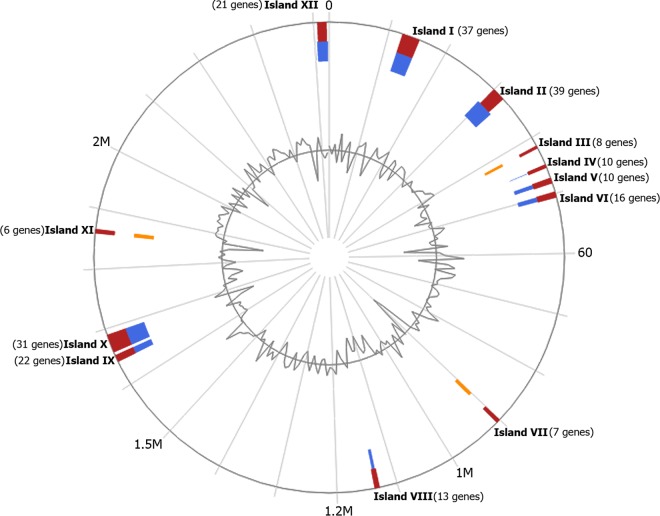
Genomic islands of strain FARPER-174 predicted by IslandViewer 4. The outer circle shows the scale line in megabase pairs. Predicted genomic islands are colored based on methods, as follows: red, integrated; blue, IslandPath-DIMOB; and orange, SIGI-HMM. The center circle represents the G+C content (%).

A total of 2,327 genes, 2,248 CDSs, 106 pseudogenes, and 79 RNA genes, which included 56 tRNAs, 19 rRNAs (5S, 16S, and 23S), 1 transfer-messenger RNA (tmRNA), and 3 noncoding RNAs (ncRNAs), were identified. Six 16S rRNA genes compared by BLASTn with the 16S rRNA database of NCBI are from 97% to 98% similar to those of *A. paragallinarum* strain NCTC 11296 (GenBank accession number NR_042932). Genomic islands were predicted by submitting the PGAAP-generated GenBank file to the IslandViewer 4 tool (18). In total, 12 genetic islands, namely, I to XII, which include 220 genes (Fig. 1), were found.

### Data availability.

The complete Avibacterium paragallinarum strain FARPER-174 genome sequence is available in GenBank under the accession number CP034110. Raw data are available in SRA under BioSample number SAMN10471982 and SRA run number SRR8506728.

## References

[1] Blackall PJ, Eaves LE, Rogers DG 1990 Proposal of a new serovar and altered nomenclature for Haemophilus paragallinarum in the Kume hemagglutinin scheme. J Clin Microbiol 28:1185–1187. 2199492 10.1128/jcm.28.6.1185-1187.1990 PMC267902

[2] Blackall PJ, Soriano-Vargas E 2017 Infectious coryza and related bacterial infections, p. 859–873. *In* Swayne D (ed), Diseases of poultry. John Wiley & Sons, Ltd, Chichester, United Kingdom.

[3] Requena D, Chumbe A, Torres M, Alzamora O, Ramirez M, Valdivia-Olarte H, Gutierrez AH, Izquierdo-Lara R, Tataje-Lavanda L, Zavaleta M, Tataje-Lavanda L, Best I, Fernández-Sánchez M, Icochea E, Zimic M, Fernández-Díaz M, FARVET Research Group 2013 Genome sequence and comparative analysis of *Avibacterium paragallinarum*. Bioinformation 9:528–536. doi:10.6026/97320630009528. 23861570 PMC3705629

[4] Horta-Valerdi G, Sanchez-Alonso MP, Perez-Marquez VM, Negrete-Abascal E, Vaca-Pacheco S, Hernandez-Gonzalez I, Gomez-Lunar Z, Olmedo-Álvarez G, Vázquez-Cruz C 2017 The genome sequence of *Avibacterium paragallinarum* Strain CL has a large repertoire of insertion sequence elements. Genome Announc 5:e00152-17. doi:10.1128/genomeA.00152-17. 28408672 PMC5391410

[5] Aguilar-Bultet L, Calderon-Copete SP, Frey J, Falquet L 2013 Draft genome sequence of the virulent *Avibacterium paragallinarum* serotype A strain JF4211 and identification of two toxins. Genome Announc 1:e00592-13. doi:10.1128/genomeA.00592-13. 23950117 PMC3744673

[6] Chen Y-C, Tan D-H, Shien J-H, Hsieh M-K, Yen T-Y, Chang P-C 2014 Identification and functional analysis of the cytolethal distending toxin gene from *Avibacterium paragallinarum*. Avian Pathol 43:43–50. doi:10.1080/03079457.2013.861895. 24188584

[7] Ho Sui SJ, Fedynak A, Hsiao WWL, Langille MGI, Brinkman FSL 2009 The association of virulence factors with genomic islands. PLoS One 4:e8094. doi:10.1371/journal.pone.0008094. 19956607 PMC2779486

[8] Bertelli C, Tilley KE, Brinkman FSL 2018 Microbial genomic island discovery, visualization and analysis. Brief Bioinform. doi:10.1093/bib/bby042. PMC6917214 29868902

[9] Dongsheng C, Hasan MS, Chen B 2014 Identifying pathogenicity islands in bacterial pathogenomics using computational approaches. Pathogens 3:36–56. doi:10.3390/pathogens3010036. 25437607 PMC4235732

[10] Falconi-Agapito F, Saravia LE, Flores-Pérez A, Fernández-Díaz M 2015 Naturally occurring β-nicotinamide adenine dinucleotide–independent *Avibacterium paragallinarum* isolate in Peru. Avian Dis 59:341–343. doi:10.1637/10969-110314-CaseR. 26473688

[11] Blackall PJ, Christensen H, Beckenham T, Blackall LL, Bisgaard M 2005 Reclassification of *Pasteurella gallinarum*, [*Haemophilus*] *paragallinarum*, *Pasteurella avium* and *Pasteurella volantium* as *Avibacterium gallinarum* gen. nov., comb. nov., *Avibacterium paragallinarum* comb. nov., *Avibacterium avium* comb. nov. and Avibacterium. Int J Syst Evol Microbiol 55:353–362. doi:10.1099/ijs.0.63357-0. 15653900

[12] Chen X, Miflin JK, Zhang P, Blackall PJ 1996 Development and application of DNA probes and PCR tests for *Haemophilus paragallinarum*. Avian Dis 40:398–407. doi:10.2307/1592238. 8790892

[13] Soriano VE, Blackall PJ, Dabo SM, Téllez G, García-Delgado GA, Fernández RP 2001 Serotyping of *Haemophilus paragallinarum* isolates from Mexico by the Kume hemagglutinin scheme. Avian Dis 45:680–683. doi:10.2307/1592911. 11569743

[14] Luna-Galaz GA, Morales-Erasto V, Peñuelas-Rivas CG, Blackall PJ, Soriano-Vargas E 2016 Antimicrobial sensitivity of *Avibacterium paragallinarum* isolates from four Latin American countries. Avian Dis 60:673–676. doi:10.1637/11398-022616-ResNote.1. 27610729

[15] Sambrook J, Russell DW 2001 Molecular cloning: a laboratory manual. Cold Spring Harbor Laboratory Press, Cold Spring Harbor, NY.

[16] Chin C-S, Alexander DH, Marks P, Klammer AA, Drake J, Heiner C, Clum A, Copeland A, Huddleston J, Eichler EE, Turner SW, Korlach J 2013 Nonhybrid, finished microbial genome assemblies from long-read SMRT sequencing data. Nat Methods 10:563–569. doi:10.1038/nmeth.2474. 23644548

[17] Tatusova T, DiCuccio M, Badretdin A, Chetvernin V, Nawrocki EP, Zaslavsky L, Lomsadze A, Pruitt KD, Borodovsky M, Ostell J 2016 NCBI Prokaryotic Genome Annotation Pipeline. Nucleic Acids Res 44:6614–6624. doi:10.1093/nar/gkw569. 27342282 PMC5001611

[18] Bertelli C, Laird MR, Williams KP, Simon Fraser University Research Computing Group, Lau BY, Hoad G, Winsor GL, Brinkman F 2017 IslandViewer 4: expanded prediction of genomic islands for larger-scale datasets. Nucleic Acids Res 45:W30–W35. doi:10.1093/nar/gkx343. 28472413 PMC5570257

